# Modeling Time to Death of Patients with Multidrug-Resistant Tuberculosis at Saint Peter’s Specialized Hospital

**DOI:** 10.34172/jrhs.2021.50

**Published:** 2021-05-19

**Authors:** Teramaj Wongel Wotale, Abiyot Negash Terefe, Jaleta Abdisa Fufa

**Affiliations:** ^1^Department of Statistics, College of Natural Science, Mettu University, Mettu, Oromia, Ethiopia; ^2^Department of Statistics, College of Natural Science, Jimma University, Jimma, Oromia, Ethiopia

**Keywords:** Hospital, Multidrug-Resistance Tuberculosis, Retrospective, Shared frailty, Time-to-Death

## Abstract

**Background:** Currently, the worldwide prevalence and incidence of multidrug-resistant tuberculosis (MDR-TB) is drastically increasing. The main objective of this study was modeling the time-to-death of patients with MDR-TB at St. Peter’s Specialized Hospital, Addis Ababa, Ethiopia, by using various parametric shared frailty models.

**Study Design:** A retrospective study design was used.

**Methods:** The study population was TB patients with MDR at St. Peter’s Specialized Hospital from January 2016 through December 2019. Exponential, Weibull, and log-normal were used as baseline hazard functions with the gamma and inverse Gaussian frailty distributions. All the models were compared based on Akaike’s Information Criteria.

**Results:** The overall median time to death was 11 months and 123 (33.5%) patients died. Patients who lived in rural areas had shorter survival time than those who lived in urban areas with an accelerated factor of 0.135 (*P*=0.002). Patients with a history of anti-TB drug consumption had a short survival time than those without such a history with an accelerated factor of 0.02 (*P*=0.001). The variability (heterogeneity) of time to death of patients in the region for the selected model (Weibull-inverse Gaussian shared frailty model) was θ=0.144 (*P*=0.027).

**Conclusion: ** The MDR-TB patients with weight gain, khat and alcohol consumption, clinical complication of pneumothorax and pneumonia, extrapulmonary TB, and history of anti-TB drug consumption as well as those who lived in rural areas had a shorter survival time, compared to others. There was a significant heterogeneity effect in the St. Peter’s Specialized Hospital. The best model for predicting the time to death of MDR-TB patients was Weibull-inverse Gaussian shared frailty model.

## Introduction

Multidrug-resistant tuberculosis (MDR-TB) is a form of tuberculosis caused by Mycobacterium tuberculosis that is resistant at least to isoniazid and rifampicin (two most powerful first-line anti-TB drugs)^1^. Currently, the majority of MDR-TB cases are due to one strain of TB bacteria called the Beijing lineage^2,3^. Currently, the worldwide prevalence and incidence rates of MDR-TB are drastically increasing^1^. The World Health Organization has estimated that there were 484,000 new cases with resistance to rifampicin, 78% of which had MDR-TB^4^.

In China, 98% of bacteriologically confirmed patients had MDR-TB^5^. Moreover, it has been reported that the incidence rate of MDR-TB is particularly high in the continent of Africa. Africa accounts for 46% of all TB cases in the world and the highest reported incidence rate of 475 cases per 100,000 people^4^. Evidence indicates the high prevalence of MDR-TB in many areas of Africa^6^. For instance, recent investigations have shown that the prevalence rates of MDR-TB in Nigeria, Zambia, Rwanda^1^, and South Africa^4^ are 54%, 9.5%, 9.4%, and 73%, respectively.

Ethiopia is also one of the 30 TB high burden countries with the highest estimated number of MDR-TB cases^7^. It accounts for 36% of all the TB cases and the highest reported incidence rate with 275 cases per 100,000 people. Findings of studies performed in some parts of Ethiopia have indicated the high prevalence of MDR-TB; for example, its prevalence rates in Oromia Region, Amhara Region, and Addis Ababa have been reported at 11.8%^8^, 15.3%^9^, and 46.3%^10^, respectively. Even though MDR-TB is treatable and curable by second-line medications, the medications are limited and extensive chemotherapy is required (up to two years of treatment)^4^. In addition, the second-line medications are less effective, more toxic, and much more expensive than the first-line anti-TB drugs^11^.

Diagnosis of drug resistance is difficult in low-resource countries^4^. Due to this worldwide issue, nearly 3 out of every 10 MDR-TB patients who start second-line MDR-TB treatments are diagnosed and about 214,000 of them die^1^. Mortality due to MDR-TB is also high in other countries around the world. For instance, 36.4% and 21.3% of MDR-TB patients died in Ukraine and Swaziland, respectively^12,13^. Furthermore, in Ethiopia, the rate of incidence and insufficient treatment outcome of MDR-TB is too high and has significant effects on efficiency of the treatment outcome. The prevalence of patients with poor treatment outcomes has increased over time from 6% per year during 2010–2012 to 12% per year during 2013-2015^5^.

 MDR-TB is one of the major health problems in Ethiopia; it is a life-long challenging disease. Despite the design of strategies to provide culture and drug susceptibility testing services, it is still affecting the lives of many people at the regional as well as national level. This disease tends to cause death mostly when it is accompanied by other diseases, like hypertension, diabetes, cardiac illness, asthma, renal disease, pneumothorax, and pneumonia. Moreover, it makes patients economically dependent on their family/household members. Hence, there is a needs to identify factors considerably related to it and give due attention to these problems to prolong the life of MDR-TB patients. In this regard, this study aimed to assess the modeling time to death of TB patients under the treatment of MDR-TB.

 Study of the survival time of patients with MDR-TB is a mechanism of overcoming the problem of health in the society (community) through identification of the risk factors associated with time to death of MDR-TB patients. Results of this study might provide evidence to the governmental and non-governmental organizations (sectors) and other concerned bodies in the development of related policies, strategies, plans, and further investigations for the control and management of the death of MDR-TB patients. In addition, it can be used as a stepping stone for further studies on MDR-TB patients.

 The structure of the article was presented as introduction, methods, results, discussion, conclusion, and references.

## Methods

###  Location of research

 The study location was St. Peter’s Specialized Hospital which was established in 1953. The St. Peter’s Hospital was formerly located in two places; the outpatient department was located in Kolfe sub-city while the Sanatorium was located in Entoto, which is the current place of Gulele sub-city, woreda 01 of Addis Ababa. This hospital is administered by the federal government and provides 24-h services to the community. In 1960, the hospital was named TB Demonstration and Training Centre and Sanatorium and become the first TB referral hospital in Ethiopia.

###  Data collection 

 The required data were extracted from follow-up charts and cards of MDR-TB patients admitted to the hospital from January 2016 to December 2019. It should be mentioned that the data collectors were healthcare professionals.

###  Study population and variables

 The study population was all MDR-TB patients who had been registered at St. Peter’s Specialized Hospital. In total, 367 patients with MDR-TB from the Hospital were included in the study. The response variable was time to death of the MDR-TB patients and time was measured by months in this study. The patients who were lost to follow-up or survived were extracted from the study. Explanatory variables were age, weight, education level, place of residence, occupation, presence of chronic diseases, clinical complication, category of MDR-TB, previous use of anti-TB drugs, and social drug use.

###  Study Design

 A retrospective study design was employed and the data were obtained from a cohort of MDR-TB patients admitted to the hospital. The time of the study was from January 2016 to December 2019, with a 48-months follow-up period. It must be noted that the present study only involved patients admitted in 2016 (from January to December). Entry of the data was considered from the date of initial administration of MDR-TB drugs until the patients developed treatment outcomes or the end of the research period was reached in December 2019. The time was measured by months in this study, and the data were analyzed in R software (version 3.5.3).

###  Inclusion and exclusion criteria

 This study included all MDR-TB patients registered from January 2016 to December 2019 for MDR-TB treatment or those who continued MDR-TB intervention. Patients whose recorded information was insufficient, either in the registration book or in the card, were excluded from the study. Besides, the patients who did not start any MDR-TB treatments were excluded as well.

###  Ethics approval and consent to participate

 Ethical approval was obtained from the Institutional Research Ethics Review Committee of the Jimma University College of Natural Sciences. A letter of support was written for the hospital that was the location of the study. The secondary data were collected without including identities (e.g., names) of the patients.

###  Statistical methods

Hazard and Kaplan-Meier estimate (KM) were used to describe the time to death of MDR-TB patients. Commonly used parametric distributions in survival analysis are the exponential, Weibull, and log-normal ^14,15^. The exponential distribution is one of the most popular parametric models due to its historical significance, mathematical simplicity, and important properties. It has a survival function which is as follows: S(𝑥)=exp⁡(-λ𝑥) for 𝑥>0. Although the exponential distribution is popular in some situations, it is restrictive in many real applications due to its functional features^16^.

The Weibull distribution has a survival function of S(𝑥)=exp⁡(-λ𝑥α) for 𝑥≥0. Here, λ>0 is a scale parameter and α > 0 is a shape parameter. The exponential distribution is a special case of the Weibull distribution when α = 1. Another frequently used distribution to model survival times is log-normal. If X has a log-normal distribution, then ln⁡(𝑥) has a normal distribution. For time-to-event data, this distribution has been popularized not just because of its relationship to the normal distribution, but since it can approximate survival times^17,18^. Similar to the normal distribution, the log-normal distribution is completely specified by two parameters, namely μ and σ^16^.

 In this study, an application of random effects model for time to death was clustered based on regions. The frailty model was used to explain the existence of the differences in the time to death among patients of different clusters (regions). Observations from the same cluster (region) are assumed to be correlated since they usually share certain unobserved characteristics, like environmental and/or genetic conditions. Ignoring the correlations among the observations may lead to incorrect standard errors of the estimates of parameters of interest.

The frailty model was used to introduce random effects in the model to account for associations and unobserved heterogeneities^19^. A shared frailty model was considered a mixed (random effects) model in survival analysis with group variation (frailty) and individual variation described by the hazard function. Shared gamma frailty with parameters ( $\frac{1}{\theta },\frac{1}{\theta }$ ) is called a one-parameter gamma distribution with variance parameter θ. With the assumption γ=δ (necessary for identifyability reasons), the two-parameter gamma distribution turns into a one parameter distribution Γ( $\frac{1}{\theta },\frac{1}{\theta }$ ). The functional form of the one-parameter gamma distribution is as follows:


(1) fUu=u1θ−1exp−u/θθ1θΓ1θ

Therefore, the variance and expectation of the frailty variable will be θ and 1, respectively.

The inverse Gaussian (inverse normal) distribution was introduced as a frailty distribution alternative to the gamma distribution by Hougaard ^20^ and has been used by Klein ^21^, and Duchateau & Janssen ^22^. The probability density function of an inverse Gaussian shared distributed random variable with parameter θ > 0 is as follows:


(2) $$fZ\left(Zi\right)=\frac{1}{\sqrt{2\pi }}Zi^{-\frac{3}{2}}\exp{\left(\frac{-\left(zi-1\right)}{2\theta zi}\right)}^{2},\theta >0,z>0$$

The mean and variance values are 1 and 𝜃, respectively. The parametric-shared frailty models were used assuming the Exponential, Weibull, and log-normal distributions for the baseline hazard function.

 Based on the model selection criteria, we compared the model using the Akaike information criterion (AIC); the smallest AIC value was the most efficient model to describe the dataset. Moreover, cox snell residual plots were used to check the goodness of fit of the model; accordingly, if the plot showed that the line related to the cox-snell residuals were nearest to the line through the origin, the model was considered well fit to the data set.

## Result s

Table 1 summarizes the descriptive summaries of baseline categorical covariates. In total, 367 MDR-TB patients were included in the study during the data collection, and 33.5% of them died. The overall median of the time to death of MDR-TB patients was 11 months [95% CI: 10, 13]. The mortality rate of MDR-TB was higher in females (17.4%) than in males (16.1%). In addition, more urban residents (25.3%) died due to MDR-TB, compared to rural residents (8.2%). The mortality rates of MDR-TB among khat users, alcohol users, smokers, and those who used a combination of any two or more social drugs were 15.3%, 3.5%, 13%, 0.3%, and 1.4%, respectively. Moreover, 21% of the MDR-TB patients were HIV-negative. The median and standard deviation [median±SD] of age and weight of patients were 39±13.44 years and 56±7.61 kg, respectively.

The multivariable survival analysis was performed supposing the exponential, Weibull, and log-normal for the baseline hazard function and the gamma and inverse Gaussian frailty distributions. The covariates which were not significant in univariable were not involved in multivariable analysis. The AIC value of the Weibull-Inverse-Gaussian model was 789.1995 which was the minimum value, compared to all the other models. Hence, the Weibull-Inverse-Gaussian model was the most efficient model to describe the dataset of patients with MDR-TB (Table 2).

A separate analysis was performed after the identification of the best model. It should be mentioned that in the analysis, a p-value of less than 5 was considered statistically significant. Based on the Weibull-Inverse-Gaussian model, the age, weight, social drug use, clinical complication, category of MDR-TB, educational level, history of anti-TB drug consumption, occupation, and place of residence of the patients were significant. Weight gain, khat and alcohol consumption, clinical complications of pneumothorax and pneumonia, extrapulmonary TB, history of anti-TB drug consumption, and residence in rural areas shortened the time to death of MDR-TB patients. On the other hand, those who had a secondary education level and diploma and a unit of age increase had a significantly prolonged survival time to death (Table 3).

An acceleration factor (Ф) greater than 1 specifies prolonging the time of death. The acceleration factors for patients with khat and alcohol consumption were 0.03 and 0.61, respectively. This implies that the khat and alcohol users had a shorter time to death, compared to non-drug users. Regarding the patients who had a clinical complication, the estimated acceleration factor of pneumothorax was estimated at 0.04 with a small *p*-value (*P*=0.003; 95%CI: 0.01, 0.33). This indicates that the patients who had no clinical complication had prolonged survival time, compared to patients with pneumothorax complication. This is similar to patients who had pneumonia with an estimated acceleration factor of 0.57 with a small *p*-value (*P*=0.023; 95%CI: 0.35, 0.93). Accordingly, it can be said that patients who had pneumonia complications had shorter survival time, compared to those who had no clinical complication (Table 3).

 Patients with extra-pulmonary had an acceleration factor of 0.09 [95%CI: 0.03, 0.26] which indicated that the patients with pulmonary MDR-TB had longer survival time in comparison with extra-pulmonary MDR-TB patients. Moreover, the patients who lived in rural areas had a shorter survival time than those who lived in urban areas (Ф=0.14; 95%CI: 0.04, 0.47). In addition, patients with secondary education level [Ф=2.31; 95%CI: 1.27, 4.23] and diploma (Ф =2.15; 95%CI: 1.09, 4.26) had prolonged survival time, compared to those with primary education level.

It was also found that the increase of age (Ф =1.02; 95%CI: 1.01, 1.03) and weight (Ф =0.95; 95%CI: 0.92, 0.98) led to a decrease in the survival time. Patients who had a history of anti-TB drug consumption (Ф =0.02) had a shorter survival time than patients with no history of anti-TB drug consumption. The estimated acceleration factor of death varied for MDR-TB patients with chronic diseases. Those patients with two or more chronic diseases (Ф =0.04; 95%CI: 0.20, 0.82) had shorter survival times than those with hypertension (Table 3).

**Table 1:** Descriptive summary of the characteristics of patients with multidrug-resistant tuberculosis (MDR-TB)

**Covariates**	**Patients**		**Deaths**		**Median in a month (95% CI)**
	**Number**	**Percent**	**Number**	**Percent**	
Gender					
Male	188	51.23	59	16.1	11 (10, 13)
Female	179	48.77	64	17.4	8 (6, 9)
Education level					
Primary	65	17.71	17	4.6	11 (9, 13)
Secondary	106	28.89	44	12	10 (9, 11)
Diploma	61	16.62	21	5.7	9 (8, 12)
Degree	56	15.26	12	3.3	10 (8, 13)
Other	79	21.53	29	7.9	8 (4, 11)
Region					
Addis Abeba	136	37.06	38	10.4	10 (8, 12)
Oromia	88	23.98	28	7.6	11 (9, 13)
SNNP	62	16.89	24	6.5	11 (10, 12)
Amhara	46	12.53	21	5.7	12 (9, 15)
Other	35	9.54	12	3.3	11 (9, 13)
Residence					
Urban	298	81.20	93	25.3	11 (10, 11)
Rural	69	18.80	30	8.2	8 (6, 9)
Occupation					
Farmer	49	13.35	16	4.4	11 (9, 12)
Employer	122	33.24	34	9.3	12 (10, 14)
Merchant	95	25.89	35	9.5	11 (7, 14)
Housewife	53	14.44	22	6.0	10 (8, 11)
Students	31	8.45	8	2.2	13 (7, 18)
Other	17	4.63	8	2.1	8 (7, 13)
Social drug use					
Non-user	149	40.60	56	15.3	10 (8, 11)
Khat use	59	16.08	13	3.5	11 (8, 12)
Alcohol use	116	31.61	48	13	11 (8, 13)
Smoker	27	7.36	1	0.3	7 (4, 9)
Two or more social drugs	16	4.36	5	1.4	10 (8, 11)
HIV status					
Negative	242	65.94	77	21.0	11 (8, 13)
Positive	125	34.06	46	12.5	11 (9, 12)
Chronic diseases					
Hypertension	124	33.79	41	11.2	11 (9, 12)
Diabetes	99	26.98	23	6.3	13 (9, 17)
Cardiac illness	62	16.89	22	6	13 (12, 14)
Asthma	35	9.54	15	4	9 (4, 10)
Renal disease	27	7.36	8	2.2	11 (8, 12)
Two or more chronic diseases	20	5.50	14	3.8	7 (5, 9)
Category of MDR-TB					
Pulmonary	306	83.38	108	29.4	10 (9, 11)
Extra-Pulmonary	61	16.62	15	4.1	12 (10, 14)
History anti-TB drug consumption					
Yes	70	19.07	35	9.5	7 (6, 8)
No	297	80.93	88	24	11 (10, 12)
Clinical complication					
No complication	121	32.97	28	7.6	11 (8, 12)
Pneumothorax	90	24.52	40	10.9	9 (7, 10)
Pneumonia	114	31.06	32	8.7	11 (10, 12)
Hemoptysis	28	7.63	16	4.4	8 (6, 9)
Corpulmonale	6	1.63	2	0.5	7 (6, 9)
Combination of any	8	2.18	5	1.4	6 (5, 8)

Source: ST, Peter’s Specialized Hospital, Ethiopia; from January 2016 to December 2019.

The value of the shape parameter in the Weibull-Inverse-Gaussian frailty model was ρ=2.47. This value is greater than unity, which indicates the shape of the hazard function is uni-modal. This means that it increases up to a point in time and then decreases. The variability (heterogeneity) in the population of clusters estimated by our best model was θ=0.14 (*P*=0.027) and the dependence within clusters was about τ=6.7%. There were differences in the death rate of patients of different Ethiopian regions.

**Figure 1:**
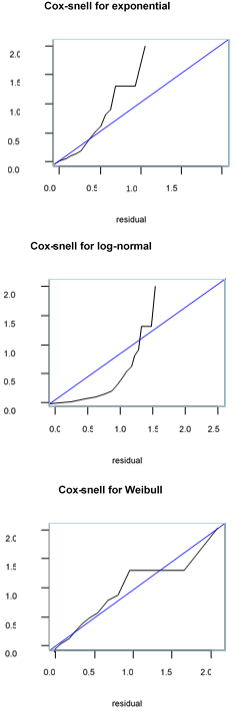
Cox-Snell residuals for parametric models

**Figure 2:**
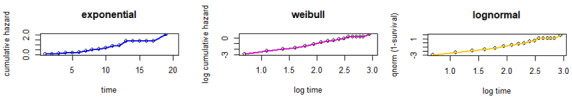
Parametric assumption testing models

The cox-snell residuals provide a unique method to study how well the model fits the data. The plot for the fitted model residuals for Weibull to our data assumed maximum likelihood estimation (Figure 1). The plot shows that the line related to the cox-snell residuals of the Weibull model was nearest to the line via the origin, meaning that this model describes the MDR-TB data set well. This outcome consolidates the result from the log cumulative hazard plot (Figure 2).

**Table 2:** Akaike information criterion values of the parametric frailty models

**Baseline distribution model **	**AIC**
Exponential	
Inverse- Gaussian	897.82
Gamma	896.18
Weibull	
Inverse- Gaussian	789.20
Gamma	791.85
Log-normal	
Inverse- Gaussian	805.40
Gamma	805.34

Source: ST, Peter Specialized Hospital, Ethiopia; from January 2016 to December 2019. AIC=Akaike information Criteria

**Table 3:** Multivariable analysis using the Weibull- inverse Gaussian frailty model

**Covariates**	**Estimates**	**SE**	***P*** **-value**	Ф ** (95% CI)**
Age (year)	0.02	0.01	0.048	1.02 (1.01, 1.03)
Weight (kg)	-0.06	0.02	0.001	0.95 (0.92, 0.98)
Residence				
Urban	Ref.			
Rural	-2.00	0.64	0.002	0.13 (0.04, 0.47)
Education level				
Primary	Ref.			
Secondary	0.84	0.31	0.006	2.31 (1.27, 4.23)
Diploma	0.77	0.35	0.027	2.15 (1.09, 4.26)
Degree	0.16	0.33	0.625	1.17 (0.61, 2.26)
Other	0.52	0.39	0.182	1.68 (0.78, 3.62)
Occupation				
Farmer	Ref.			
Employer	-0.13	0.30	0.658	0.88 (0.48, 1.58)
Merchant	-0.73	0.39	0.064	0.48 (0.22, 1.04)
Housewife	-0.62	0.34	0.069	0.54 (0.28, 1.04)
Student	-0.92	0.47	0.051	0.39 (0.16, 1.01)
Other	1.18	0.46	0.011	3.24 (1.31, 7.98)
Social drug				
Non-use	Ref.			
Khat use	-3.38	1.01	0.002	0.03 (0.00, 0.29)
Alcohol use	-0.50	0.24	0.035	0.61 (0.38, 0.96)
Smoker	-0.28	0.38	0.477	0.76 (0.35, 1.61)
≥2 social drugs	-0.01	0.59	0.990	0.99 (0.31, 3.15)
Chronic diseases				
Hypertension	Ref.			
Diabetes	-0.32	0.29	0.285	0.73 (0.40, 1.30)
Cardiac illness	0.35	0.31	0.269	1.42 (0.76, 2.62)
Asthma	0.25	0.33	0.461	1.28 (0.67, 2.46)
Renal diseases	0.61	0.49	0.212	1.85 (0.70, 4.84)
≥2 chronic diseases	-0.91	0.36	0.012	0.40 (0.19, 0.82)
Clinical				
No complication	Ref.			
Pneumothorax	-3.17	1.06	0.003	0.04 (0.01, 0.33)
Pneumonia	-0.57	0.25	0.023	0.57 (0.35, 0.93)
Hemoptysis	-0.46	0.33	0.162	0.63 (0.34, 1.20)
Corpulmonale	0.21	0.64	0.747	1.23 (0.35, 4.32)
Combination of any	-0.44	0.61	0.472	0.64 (0.19, 2.15)
Category				
Pulmonary	Ref.			
Extra-pulmonary	-2.36	0.51	0.001	0.09 (0.03, 0.26)
Previous use of anti-TB drugs				
No	Ref			
Yes	-3.49	1.00	0.001	0.02 (0.47, 0.78)

θ=0.14, (*P*=0.027), τ=0.07, λ=0.04, SE=0.04, ρ=2.47, SE=0.18, Akaike information criterion=789.20 θ: Variance random effect, τ: Kendall's Tau, λ: lambda ρ: shape, Ф: accelerated factor, SE: standard error Source: St. Peter’s Specialized Hospital, Ethiopia; from January 2016 to December 2019.

**Figure 3:**
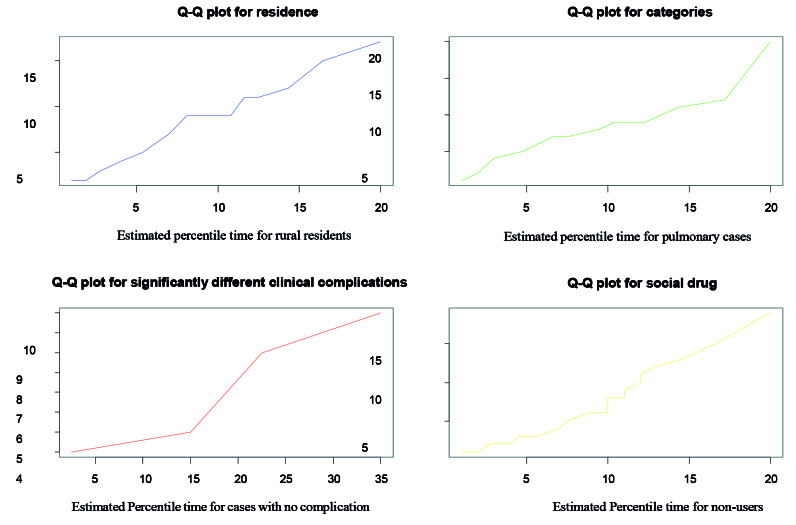
Quantile-quantiles plot (Q-Q plot) to check the adequacy of the accelerated failure time models

A quantile-quantiles plot was made to check if the accelerated failure time provided an adequate fit to the data by using two different groups of observation. Moreover, the adequacy of the model was checked using different significant variables. The accelerated failure time appears to be the best way to describe the survival time of the MDR-TB dataset (Figure 3).

## Discussion

There was a cluster (frailty) effect based on the region, considering that patients coming from the same region shared similar risk factors. In addition, it was found that non-drug users had a longer survival time than those who used drugs. Results of the current study are consistent with those of other studies conducted in Ethiopia^23–26^, Botswana^27^, and Sudan^28^. This might be due to the fact that unhealthy habits, such as consuming alcohol, chewing khat, and smoking, expose the patients to many diseases, especially those with serious infections, like MDR-TB. Such habits facilitate super-imposed infections and compromise the immunity of the patients and shorten their survival time. They might also harm the breathing organs of the patients, including their lungs and respiratory systems.

Based on the results, MDR-TB patients with different clinical complications (e.g., pneumonia and pneumothorax complications) had shorter survival time, compared to MDR-TB patients with non-clinical complications. This was in line with the findings of some studies carried out in Ethiopia^23,29^ - ^32^. The similarity might be due to the fact that the pre-existing infections can be further complicated by the newly emerging diseases. These newly emerging diseases decrease the immunity of patients, which can shorten their survival time.

Findings of this study also revealed that the MDR-TB patients who developed a combination of two or more chronic diseases had a shorter survival time than the reference group. This finding was consistent with those of previous studies performed in Ethiopia^26^, State of Georgia^33^, and Southeastern Mexico^34^. This might be due to the fact that co-morbidity is the risk factor for different diseases, like infectious and non-infectious ones, which could shorten the survival time of the patients.

In this study, it was found that age had a minimal effect on the mortality of MDR-TB patients. This finding was in line with those of other studies conducted in Ethiopia^26,29,32^. Accordingly, the increase in the age of patients led to a decrease in their survival time. The results also indicated that the patients who lived in rural areas had shorter survival time than those who lived in urban areas. This was consistent with the findings of previous studies conducted in Sudan^28^, East Shoa, and Ethiopia^24^. This might be due to the reason that people who live in rural areas are less aware of health issues; in addition, they might have lower health-seeking behaviors. Besides, even those who had awareness or more health-seeking behaviors might not have had access to health centers.

In this study, the patients with secondary education level and diploma had longer survival time in comparison with the reference group. This finding was in line with those of the studies performed in Ethiopia^25^ and Lithuania^35^. This could be due to the lack of awareness of people with lower levels of education about their health issues. Moreover, they might have had no access to media, such as social media.

## Limitations

 In this paper, the effects of MDR-TB interventions as well as nutritional and laboratory information (drug susceptibility test results and adherence status) were not considered which is the limitation of the study in general and a prediction model in particular.

## Conclusion

 The variables significantly influencing the MDR-TB patients were age, weight, social drug use, clinical complication, category of MDR-TB, education level, history of anti-TB drug consumption, occupation, and place of residence of the patients. The MDR-TB patients with weight gain and khat and alcohol use, history of anti-TB drug consumption, clinical complication of pneumothorax and pneumonia, extrapulmonary TB, and rural residency had shorter survival time, compared to others. On the other hand, those with a secondary education level and diploma and a unit of age increase had prolonged survival time. There were differences in the death rate of MDR-TB patients in the regions at Saint Peter’s Specialized Hospital. Weibull-Inverse-Gaussian shared frailty model was the most efficient model to describe the MDR-TB patients.

## Acknowledgment

 The authors gratefully acknowledge St. Peter’s Specialized Hospital for the provision of the data.

## Conflict of interest

 The authors declare that there was no conflict of interest in this study.

## Funding

 The study was funded by Jimma University (JU). The JU only granted the budget and was not involved in the design of the study, collection, analysis, interpretation of data, and writing the manuscript.

## Highlights

- Mortality rate of multidrug-resistant tuberculosis (MDR-TB) was 33.5%.
- History of anti-TB drug consumption shortens the survival time of MDR-TB patients.
- There was a significant regional difference in terms of the time to death of MDR-TB patients in Saint Peter’s Specialized Hospital.

## References

[1] World Health Organization. Global tuberculosis report 2018. Geneva: WHO; 2018.

[2] Parwati I, van Crevel R, van SD (2010). Possible underlying mechanisms for successful emergence of the Mycobacterium tuberculosis Beijing genotype strains. Lancet Infect Dis.

[3] Stoffels K, Allix-be C, Groenen G, Wanlin M, Berkvens D, Mathys V (2013). From Multidrug- to Extensively Drug-Resistant Tuberculosis : Upward Trends as Seen from a 15-Year Nationwide Study. PloS One.

[4] World Health Organization. Global status report on alcohol and health 2019. Geneva: WHO; 2019.

[5] Alene KA, Viney K, Mcbryde ES, Tsegaye AT, Clements ACA (2017). Treatment outcomes in patients with multidrug-resistant tuberculosis in north-west Ethiopia. Trop Med Int Health.

[6] Meskel DW, Abate G, Lakew M, Goshu S, Aseffa A (2008). Anti-tuberculosis drug resistance among retreatment patients seen at St Peter Tuberculosis Specialized Hospital. Ethiop Med J.

[7] Workicho A, Kassahun W, Alemseged F (2017). Risk factors for multidrug-resistant tuberculosis among tuberculosis patients : a case-control study. Infect Drug Resist.

[8] Mehari K, Asmelash Ts, Hailekiros H, Wubayehu T, Godefay H, Araya T (2019). Prevalence and factors associated with multidrug-resistant tuberculosis (MDR-TB) among presumptive MDR-TB patients in Tigray Region, Northern Ethiopia. Can J Infect Dis Med Microbiol.

[9] Abate D, Taye B, Abseno M, Biadgilign S (2012). Epidemiology of anti-tuberculosis drug resistance patterns and trends in tuberculosis referral hospital in Addis Ababa, Ethiopia. BMC Res Notes.

[10] Biadglegne F, Mulu A, Rodloff AC, Sack U (2014). Diagnostic performance of the Xpert MTB/RIF assay for tuberculous lymphadenitis on fine needle aspirates from Ethiopia. Tuberculosis.

[11] Millard J, Ugarte-Gil C, Moore DA (2015). Multidrug resistant tuberculosis. BMJ.

[12] Verdecchia M, Keus K, Blankley S, Vambe D, Ssonko C, Piening T (2018). Model of care and risk factors for poor outcomes in patients on multi-drug resistant tuberculosis treatment at two facilities in eSwatini (formerly Swaziland), 2011 – 2013. PLoS One.

[13] Aibana O, Bachmaha M, Krasiuk V, Rybak N, P Flanigan T, Petrenko V (2017). Risk factors for poor multidrug-resistant tuberculosis treatment outcomes in Kyiv Oblast Ukraine. BMC Infect Dis.

[14] Cantor, Alan B. SAS Survival Analysis Techniques for Medical Research, Second Edition. Cary, NC: SAS Institute Inc; 2003.

[15] Allison, Paul D. Survival Analysis Using SAS: A Practical Guide, Second Edition. Cary, NC: SAS Institute Inc; 2010.

[16] Klein, John P, Moeschberger, Melvin L. Survival Analysis: Techniques for censored and truncated data. Springer; 2003.

[17] Horner RD (1987). Age at Onset of Alzheimer’s Disease: Clue to the Relative Importance of Etilogic Factors. Am J Epidemiol.

[18] Feinleib M (1960). A method of Analyzing Log Normally Distributed Survival Data with Incomplete Follow-Up. J Am Stat Assoc.

[19] Wintrebert CMA. Statistical modelling of repeated and multivariate survival data. Leiden University; 2007 [cited 10 March 2021]. Available from; https://scholarlypublications.universiteitleiden.nl/handle/1887/11456.

[20] Hougaard P (1984). Life table methods for heterogeneous populations: distributions describing the heterogeneity. Biometrika.

[21] Klein JP (1992). Semiparametric estimation of random effects using the Cox model based on the EM algorithm. Biometrics.

[22] Duchateau L, Janssen P. The frailty model. Springer; 2007.

[23] Woya AA, Tekile AK, Basha GW. Spatial frailty survival model for multidrug-resistant tuberculosis mortality in Amhara Region, Ethiopia. Tuberc Res Treat; 2019. 8742363. 10.1155/2019/8742363 PMC6332868 30693105

[24] Desissa F, Workineh T, Beyene T (2018). Risk factors for the occurrence of multidrug-resistant tuberculosis among patients undergoing multidrug-resistant tuberculosis treatment in East Shoa, Ethiopia. BMC Public Health.

[25] Mulisa MG, Workneh T, Hordofa N, Suaudi M, Abebe G, Jarso G (2015). Multidrug-resistant Mycobacterium tuberculosis and associated risk factors in Oromia Region of Ethiopia. Int J Infect Dis.

[26] Fantaw D, Feyissa M, Hamid S, Sibeshi W (2018). Assessment of the Survival Status and Risk Factors for the Mortality among Multidrug Resistant Tuberculosis Patients at Adama and Bishoftu General Hospitals, Oromia, Ethiopia: A Retrospective Cohort Study. Adv Pharmacoepidemiol Drug Saf.

[27] Zetola NM, Modongo C, Kip EC, Gross R, Bisson GP, Collman RG (2012). Alcohol use and abuse among patients with multidrug-resistant tuberculosis in Botswana. Int J Tuberc Lung Dis.

[28] Ali MH, Alrasheedy AA, Hassali MA, Kibuule D, Godman B (2019). Predictors of multidrug-resistant tuberculosis (MDR-TB) in Sudan. Antibiotics.

[29] Mitku AA, Dessie ZG, Muluneh EK, Workie DL (2016). Prevalence and associated factors of TB/HIV co-infection among HIV Infected patients in Amhara region, Ethiopia. Afr Health Sci.

[30] Girum T, Tariku Y, Dessu S (2017). Survival status and treatment outcome of multidrug resistant tuberculosis (MDR-TB) among patients treated in treatment initiation centers (TIC) in South Ethiopia: a retrospective cohort study. Ann Med Health Sci Res.

[31] Limenih YA, Workie DL (2019). Survival analysis of time to cure on multi-drug resistance tuberculosis patients in Amhara region, Ethiopia. BMC Public Health.

[32] Molalign S, Wencheko E (2015). Risk factors of mortality in patients with multi-drug resistant TB. Ethiop J Health Dev.

[33] Magee MJ, Foote M, Maggio DM, Howards PP, Narayan KV, Blumberg HM (2014). Diabetes mellitus and risk of all-cause mortality among patients with tuberculosis in the state of Georgia, 2009–2012. Ann Epidemiol.

[34] Pérez-Navarro LM, Fuentes-Domínguez FJ, Zenteno CR (2015). Type 2 diabetes mellitus and its influence in the development of multidrug resistance tuberculosis in patients from southeastern Mexico. J Diabetes Complications.

[35] Ali AO, Prins MH (2017). Disease and treatment-related factors associated with tuberculosis treatment default in Khartoum State, Sudan: a case-control study. East Mediterr Health J.

